# The role of gambling type on gambling motives, cognitive distortions, and gambling severity in gamblers recruited online

**DOI:** 10.1371/journal.pone.0238978

**Published:** 2020-10-06

**Authors:** Sasha Mathieu, Servane Barrault, Paul Brunault, Isabelle Varescon

**Affiliations:** 1 Laboratoire de Psychopathologie et Processus de Santé, Université de Paris, Boulogne-Billancourt, France; 2 Laboratoire QualiPsy EE1901, Département de Psychologie, Université de Tours, Tours, France; 3 CHRU de Tours, Centre de Soins d’Accompagnement et de Prévention en Addictologie (CSAPA 37), Tours, France; 4 CHRU de Tours, Equipe de Liaison et de Soins en Addictologie & Clinique Psychiatrique Universitaire, Tours, France; 5 UMR 1253, iBrain, Université de Tours, Inserm, Tours, France; Universidad de Granada, SPAIN

## Abstract

The recent literature shows that the type of gambling practiced influences problem gambling. This study was aimed at investigating the factors associated with gambling type, including gambling severity, gambling motives, and cognitive distortions. A total of 291 regular male gamblers (229 skill gamblers and 62 mixed gamblers, i.e., those who play at least one game of chance and one skill game) were recruited online and assessed for gambling severity (South Oaks Gambling Screen), gambling motives (Gambling Motives Questionnaire-Financial), cognitive distortions (Gambling-Related Cognition Scale), and psychological distress (Hospital Anxiety and Depression Scale). After controlling for the number of games played and psychological distress, we found that gambling type was significantly associated with gambling severity. Moreover, controlling for psychological distress showed that gambling type was also significantly associated with coping motives and interpretative bias. First, mixed gamblers had higher severity scores and higher coping motivation than skill gamblers; second, skill gamblers seemed more at risk of developing interpretative bias. Thus, the gamblers presented different psychological, motivational, and cognitive profiles according to gambling type, indicating that different clinical interventions may be relevant. Working on coping motives and anxiety and depression symptoms with an abstinence purpose would be more suitable for mixed gamblers. Indeed, working on these points could lead to the gambler reducing or eventually ceasing gambling, as the need to regulate negative emotions through gambling behavior would fade in parallel. Gambling type, psychological distress, gambling motives, and cognitive distortions should be taken into consideration systematically in clinical interventions of patients with plural and mixed practice of games.

## Introduction

While gambling is a leisure activity perceived as a source of entertainment for the majority of gamblers, this behavior can become problematic for some, with the experience of craving, loss of control over behavior, and maintenance despite the existence of negative consequences [1]. Although the etiology of pathological gambling is complex and multifactorial, several studies have identified gambling motives, cognitive distortions, and emotional states, respectively, as factors involved in the development and maintenance of gambling severity [2–6].

Motivation is characterized by the presence of internal and/or external forces that produce the onset, direction, persistence, and intensity of a given behavior [7]. Although the concept of motivation is not only related to gambling, it is a central concern in gambling practice because it is associated with the commitment and investment in gambling behavior. Although money is an inherent component of gambling, other gambling motives can nevertheless exist independently of or co-exist with the expectation of winning money through gambling activities [8]. Indeed, the literature has highlighted many motivations for gambling, such as playing for pleasure, for fun, avoiding boredom, escaping repetitive or intrusive negative affects, for socializing, for excitement, or to compete with others [9–12]. The most frequently validated and cited motivational model in the international literature initially presents three gambling motives: enhancement (i.e., increasing positive emotions), coping (i.e., reducing or avoiding negative emotions), and social (i.e., increasing social affiliation) [13], to which financial motivation (i.e., winning money) was later added [14]. On the one hand, the financial motive cannot be dissociated from this type of game, and on the other hand can lead to losses that can be fueled by cognitive distortions such as the illusion of control [15]. The recent integrative study of Barrada and colleagues [10], aiming to clarify the factor structure underlying a pool of motives for gambling from two widely used gambling motivation assessment tools (the Gambling Motives Questionnaire–Financial [GMQ-F], and the Reasons for Gambling Questionnaire [RGQ), also identified the first three factors (social, financial, fun-/thrill-related) and grouped positive and negative reinforcement as a fourth one (affect regulation factor).

Another important variable strongly associated with problem gambling is the existence of cognitive distortions (i.e., the existence of irrational beliefs and erroneous perceptions about gambling and luck). The association between cognitive distortions and problem gambling is well-documented in the literature [16, 17]: gambling-related cognitions are indeed important predictors of disordered gambling and are related to prognosis [18]. According to the current literature, gambling-related cognitions encompass different types of phenomena [19], including the tendency to perceive patterns or streaks more frequently in random series of gambling outcomes, the tendency to perceive causal connections in coincidental co-occurrences of environmental cues and gambling outcomes, and the overestimation of the degree of personal control over gambling outcomes. Several models have been proposed for assessing cognitive distortions [20–23], including that of Raylu and Oei, one of the most widely used in the literature. This model includes beliefs related to gambling that have been identified irrational, as well as items related to beliefs about oneself in relation to gambling. It also assesses a wide range of cognitive distortions, i.e., the five significant gambling-related cognitive distortions identified. The main cognitive distortions listed in the model of Raylu and Oei, who designed the Gambling-Related Cognition Scale (GRCS), are gambling expectancies (i.e., expectations of the effects of gambling in terms of pleasure, relief, hope and other feelings of personal utility that may be derived from the game), illusion of control (i.e., perception of controlling the outcome of the game), predictive control (i.e., perception of predicting the outcome of the game), inability to stop gambling (i.e., perception of being unable to resist an irrepressible desire to gamble), and interpretative bias (i.e., an attributional belief in which successes are attributed to oneself and failures to external factors) [20].

While cognitive distortions share a close connection with gambling motives [17] (for example, coping and financial motives were identified as being the most significant predictors of cognitive distortions in a population of male poker gamblers [17]), it seems that they can also be related with the gambler’s psychological distress, which refers to the feelings of negative affects such as stress, anxiety, and depression [1, 24]. The literature has demonstrated that gambling modalities or gambling types re important factors to consider when studying problem gambling. However, few studies have investigated the factors associated with gambling type by simultaneously taking into account gambling motives, gambling-related cognitions, and gambling severity. Thus, on the one hand, the gambler’s psychological distress tends to change depending on the gambling intensity, and on the other hand, on the game’s outcome. In fact, frequent comorbidities have been found between pathological gambling and emotional distress such as anxiety and depression symptoms [3, 25]. Although there is no consensus about the order of emergence of these disorders, some authors have indicated that anxiety and depression symptoms (as well as anxiety and mood disorders considered as categorical diagnoses) constitute risk factors in the development of gambling severity [25, 26]. However, this does not alter the fact that pathological gambling causes or reinforces initially present anxiety and/or depression symptoms [27]. Therefore, pathological gambling is a risk factor for the emergence of anxiety and depression symptoms as well as a way of coping with unpleasant and negative affect [13, 28, 29].

As described above, gambling motives are associated with the development of erroneous beliefs [15, 17], in that gambling motives and cognitive distortions are directly and indirectly involved in the development of gambling severity [1, 16, 17]. The literature has also shown that playing in order to regulate negative feelings is more present as a coping motive among problem gamblers. Although these variables appear to be closely related, we may assume that the strength of the association between these variables may differ depending on the type of gambling modality.

Differences in the psychological and psychopathological profiles of gamblers with problematic gambling activity can be observed depending on the type of games played or the types of gambling modalities [30–32]. Games can currently be classified according to whether the rewards associated with the gambling activity are immediate or delayed [33], depending on the level of arousal provided by the game played [10], or according to the presence or absence skills [34, 35]. Some means of classifying games overlap in the sense that skill games generally provide high arousal while chance games provide low arousal [10, 19]. Gamblers using gambling to escape or avoid negative affect usually orient towards games of luck, whereas gamblers using gambling to upregulate positive emotions usually choose skill games that provide sensations, excitement, and arousal [30, 36]. Thus, gambling motives, such as gambling for experience enhancement or as a way of coping (two sides of affect regulation), seem to depend on gambling type [10]. This difference in motivation can subsequently lead to differences in cognitive distortions (in terms of nature and/or intensity) and gambling severity. In addition, engaging in several types of games seems to be frequent, particularly among problem gamblers [36–38], leading researchers to take an interest in the mixed gamblers category, in which both skill and chance games are practiced. According to the current literature on motivational, cognitive, and emotional variables, it is necessary to differentiate gamblers depending on gambling activity first because they each constitute a specific population, and second because they can have different characteristics from gamblers moving towards only one type of game. This highlights the need to obtain information on each type of gambler to think about prevention actions and to offer appropriate treatment.

Taking into consideration the reality of gambling practices [36, 38], the present study was aimed at comparing skill and mixed gamblers in terms of gambling severity, gambling motives, and cognitive distortions, while taking into account psychological distress and the number of games played. We investigated two hypotheses: first, that mixed gamblers present a higher psychological distress score than skill gamblers, and second, that gambling type is associated with gambling severity, gambling motives, and cognitive distortions.

## Materials and methods

### Participants and procedure

Participants were recruited through online gambling forums (betclever, Club Poker). Once the agreement of the administrators was received, the same announcement was published on these two forums, briefly presenting the research and its objectives. Interested members were invited to click on the LimeSurvey® link to access the online scales and questionnaires (preceded by an information note and a consent form). All participants were at least 18 years old, fluent French speakers, and had regular gambling practice (i.e., at least once per week). This criterion of regularity has been used in previous studies [2, 17, 30]. In addition, participants were not undergoing treatment for a gambling problem. Ethical approval was obtained from the Research Committee of Paris University (IRB number: 20162200001072) for its realization, and before taking part, all participants provided their written informed consent.

A total of 291 male regular gamblers were included in the study. The absence of women in our sample will be discussed within the limits of the study. The participants were divided into two groups according to the type of games played: skill gamblers who played only skill games (n = 229, 78.7%), and mixed gamblers who play both skill games and games of luck (n = 62, 21.3%).

### Measures

#### Sociodemographic and gambling data

Participants were assessed for age, marital status, level of education, household composition, professional activity, socio-professional category, and games played using an 11-item questionnaire constructed for this study.

#### Gambling severity

Gambling severity was assessed with the 20 items (e.g., “When you gamble, how often do you go back another day to win back money you lost?”) from the French validated version of the South Oaks Gambling Screen (SOGS [39, 40]). A score of ≤2 represents the absence of problem gambling, a score of 3 or 4 defines a problematic use of gambling, while a score of ≥5 corresponds to probable pathological gambling. However, consistent with previous studies [32, 36], we used a gambling severity dichotomy: scores of 0–2 indicated no problem gambling and scores of ≥3 suggested problem gambling, which includes both at-risk and probable pathological gamblers. As almost no mental health problem is categorical [41], the dimensional score was used in the statistical analysis, and the categorical score was only used to describe the two subsamples. According to the literature, gambling behavior evolves over time, so only the current assessment of a gambling problem was used and the lifetime assessment was removed. Cronbach’s alpha for the total scale (α = .72) was higher than .70, indicating good reliability [42].

#### Gambling motives

Gambling motives were measured with the French validated version of the GMQ-F [3, 14]. This tool is an improved version of the GMQ [13], which was directly adapted from the Drinking Motives Questionnaire [43]. Initially, the GMQ only measured three types of motivation: enhancement (five items, e.g., “Because it’s exciting”), coping (five items, e.g., “Because it helps when you are feeling nervous or depressed”), and social (five items, e.g., “Because it makes a social gathering more enjoyable”). Subsequent studies showed the importance of the financial aspect in gambling, thus a fourth dimension of financial motives (nine items, e.g., “Because winning would change your lifestyle”) was added and assessed [14], resulting in the GMQ-F. As item 9 of the social motives subscale posed a problem during the French validation of this tool, it was deleted, leading to a model with 23 items. These were scored on a 4-point Likert scale ranging from 1 (never or almost never) to 4 (almost always or always). Regarding Nunnally’s criterion (1978), Cronbach’s alpha coefficients were satisfactory for enhancement (α = .80) and coping (α = .76), and slightly below the threshold set at .70 for the social (α = .65) and financial subscales (α = .67) [42].

#### Cognitive distortions

Cognitive distortions were measured with the 23 items from the French validated version of the GRCS [20, 44]). Items are grouped into five subscales: gambling expectancies (four items, e.g., “Gambling makes things seem better”), illusion of control (four items, e.g., “Specific numbers and colors can help increase my chances of winning”), predictive control (six items, e.g., “I have some control over predicting my gambling wins”), inability to stop gambling (five items, e.g., “It is difficult to stop gambling as I am so out of control”), and interpretative bias (four items, e.g., “Relating my losses to bad luck and bad circumstances makes me continue gambling”). These were scored on a 7-point Likert scale ranging from 1 (strongly disagree) to 7 (strongly agree). Regarding Nunnally’s criterion (1978), Cronbach’s alpha coefficients were acceptable for inability to stop gambling (α = .83) and illusion of control (α = .73), and lower for gambling-related expectancies (α = .59), predictive control (α = .54), and interpretative bias (α = .54) [42].

#### Psychological distress

Psychological distress was assessed using the French version of the Hospital Anxiety and Depression Scale (HADS [45, 46]). This is a 14-item self-report scale, seven of which relate to anxiety (e.g., “Worrying thoughts go through my mind”) and the other seven to depression (e.g., “I feel as if I have slowed down”). Items were scored 0–3. Although the literature highlights the existence of a bidimensional structure, the HADS does not provide good separation between anxiety and depression [47]. Thus, we used the HADS total score to obtain information on the participants’ overall psychological distress. Cronbach’s alpha for the scale was .83, which indicates good internal consistency [42].

### Statistical analysis

All data were analyzed with SPSS version 21 and were tested with a two-sided significance level of .05. To conduct relevant statistical analyses, we performed a skewness test: scores obtained for gambling severity, gambling motives, cognitive distortions, and psychological distress were between -1.96 and +1.96, suggesting compatibility with the realization of parametric statistics [48, 49]. First, univariate analyses (Student’s *t*-test and chi-square test) were carried out to describe and compare skill gamblers and mixed gamblers. Second, multivariable analyses were conducted (multiple linear regressions) on the whole sample to determine whether gambling type was associated with gambling severity, gambling motives, and cognitive distortions. To control the risk of being wrong for all the tests carried out, we adjusted the *p*-values by taking the confounding factors into account (i.e., the psychological distress and the number of games played). Thus, the results reported come from two models: one unadjusted and the other adjusted by controlling the confounding factors.

## Results

### Sociodemographic and gambling data

Table 1 details the sociodemographic data. Descriptive analyses revealed that gamblers were mainly higher education graduates (68.7%), employed (64.9%), married or in a relationship (47.1%), mostly without children (64.6%), and were 34.0 years old on average (*SD* = 10.2). Statistical analyses (Student’s *t*-test and chi-square test) showed no significant differences in terms of sociodemographic characteristics between skill and mixed gamblers (all, *p* ≥ .603).

**Table 1:** Sociodemographic characteristics of skill and mixed gamblers.

	Total (N = 291) M (SD)	Skill gamblers (n = 229) M (SD)	Mixed gamblers (n = 62) M (SD)	*p*
**AGE**	33.99 (10.19)	34.49 (10.55)	34.33 (8.85)	.991
	n (%)	n (%)	n (%)	
EDUCATION				.629
***<High school degree***	33 (11.3)	28 (12.2)	5 (8.1)	
***High school degree***	58 (19.9)	46 (20.1)	12 (19.4)	
***>High school degree***	200 (68.7)	155 (67.7)	45 (72.6)	
**EMPLOYMENT STATUS**				.997
***Employed***	189 (64.9)	149 (65.1)	40 (64.5)	
***Unemployed***	51 (17.5)	40 (17.5)	11 (17.7)	
***Inactive***	51 (17.5)	40 (17.5)	11 (17.7)	
**MARITAL STATUS**				.603
***Single***	134 (46.0)	107 (46.7)	27 (43.5)	
***Married/in a relationship***	137 (47.1)	108 (47.2)	29 (46.8)	
***Separated***	20 (6.9)	14 (6.1)	6 (9.7)	
**CHILDREN**				.663
***Yes***	103 (35.4)	77 (33.6)	26 (41.9)	
***No***	188 (64.6)	152 (66.4)	36 (58.1)	

M: Mean; SD: standard deviation.

The prevalence of problem gambling was 30% (n = 69) in skill gamblers and 46.8% (n = 29) in mixed gamblers: the prevalence levels were not significantly higher in mixed gamblers (χ^2^ = 2.51; *p* = .113).

In our sample, 15.8% of participants played online games exclusively, while 3.4% played exclusively land-based games. The majority of gamblers recruited played both on the Internet and in live (casinos and tobacconists). In addition, most of the participants (n = 229, 78.7%) reported playing only skill games (poker, sports betting, horses betting, blackjack), and no one reported playing only games of luck (scratch cards, slot machines, roulette, lottery). However, some participants (n = 62, 21.3%) indicated that they played both games of skill and games of chance. Skill gamblers mainly played poker and sports betting, whereas mixed gamblers mainly played poker, scratch cards, sports betting, roulette, and slot machines (Table 2). Among the skill gamblers, 44.5% played at least two games with a strategy aspect (mixed gamblers, by definition, all played at least two types of games).

**Table 2:** Gamblers’ distribution by gambling type.

	Skill gamblers n (%)	Mixed gamblers n (%)
**Land-based poker**	166 (72.5)	51 (82.3)
**Online poker**	207 (90.4)	53 (85.5)
**Land-based sports betting**	48 (21.0)	19 (30.6)
**Online sports betting**	81 (35.4)	31 (50.0)
**Blackjack**	20 (8.7)	17 (27.4)
**Land-based horse betting**	12 (5.2)	13 (21.0)
**Online horse betting**	9 (3.9)	5 (8.1)
**Scratch cards**	-	32 (51.6)
**Roulette**	-	25 (40.3)
**Slot machines**	-	18 (29.0)
**Lottery**	-	4 (6.5)

**Note:** The distinction between online and land-based gambling was not made for blackjack, scratch cards, roulette, and slot machines because the law in France allows online practice only for poker, sports betting, and horses betting.

#### Gambling severity and number of games played

The number of games played explained the gambling severity significantly (t = 2.815; *p* = .005). To dissociate the effect of the number of games played from playing different types of games, the number of games played was first included as a confounding factor in the model aimed at predicting gambling severity.

#### Gambling type and psychological distress

Mixed gamblers presented significantly higher HADS total scores (*t* = -2.63; *p* = .009; Cohen’s *d* = .36) than skill gamblers. Based on these results, psychological distress was included as a potential confounder in all regression analyses performed to limit bias in the analysis of the link between gambling type and the variables studied. As psychological distress can also be higher due gambling severity, we conducted regressions aimed at explaining gambling severity with and without this covariate.

#### Factors associated with gambling type

To clarify the association between gambling type and gambling severity, regressions were carried out, with gambling type as the independent variable, and the number of games played and psychological distress were introduced respectively into the model as adjustment variables. A third regression model aimed at explaining gambling severity was carried out without introducing psychological distress as a covariate. Statistical analyses revealed a significant predictor effect of gambling type on gambling severity when the model was adjusted for the number of games played (η^2^ = .015; adjusted *p* = .038) and for psychological distress (η^2^ = .021; adjusted *p* = .013), with a larger effect size, although remaining small, when the model was not adjusted for psychological distress (η^2^ = .040; adjusted *p* = .001). Moving from skill to mixed gamblers led to a significant increase in gambling severity, especially when the model was adjusted for the number of games played and psychological distress (confounding factors). Multiple regressions were also conducted to determine whether the gambling type (independent variable) could predict gambling motives and cognitive distortions when controlling for psychological distress. The multiple regressions revealed a significant predictor effect of gambling type on coping motives (η^2^ = .015; adjusted *p* = .036) and interpretative bias (η^2^ = .014; adjusted *p* = .040). When controlling for psychological distress (HADS total score), moving from skill to mixed gamblers also led to a significant increase in coping motives and interpretative bias (Table 3).

**Table 3:** South Oaks Gambling Screen, Gambling Motives Questionnaire–Financial, and Gambling-Related Cognitions Scale scores as a function of gambling type.

		Skill (n = 229) M (SD)	Mixed (n = 62) M (SD)	Éta^2^	*p*-value	Adjusted *p*-value^a^
**South Oaks Gambling Screen**						
	Total score	2.1 (2.4)	3.4 (3.3)	.021	.001***	.013*
**Gambling Motives Questionnaire–Financial**						
	Enhancement	13.4 (3.7)	14.1 (3.5)	.005	.187	.226
	Social	5.6 (1.8)	6.0 (1.2)	.009	.084	.098
	Coping	8.3 (2.9)	9.6 (3.6)	.015	.003**	.036*
	Financial	17.6 (4.6)	17.8 (5.0)	< .001	.713	.687
**Gambling-Related Cognitions Scale**						
	Gambling-related expectancies	14.2 (4.5)	14.0 (5.0)	.002	.808	.480
	Illusion of control	5.6 (3.1)	6.7 (4.6)	.006	.028*	.180
	Predictive control	16.0 (5.7)	15.2 (6.2)	.004	0.35	.302
	Inability to stop gambling	12.5 (6.6)	14.3 (8.0)	.002	.077	.416
	Interpretative bias	15.7 (4.7)	14.6 (5.8)	.014	.096	.040*

^a^ Adjusted for the overall psychological distress score.

*p < .05;

**p < .01;

***p < .001.

## Discussion

In this study, we investigated two hypotheses: first, that mixed gamblers present a higher psychological distress score than skill gamblers, and second, that gambling type is a predictor of gambling severity, gambling motives, and cognitive distortions. Our study sheds light on the specificity of mixed gambling, which was associated with higher gambling severity, higher coping motives, and higher interpretative bias. Moreover, our study shows that mixed gambling is associated with greater psychological distress. Finally, we found that a relation between gambling type and gambling severity exists, as does the association between the number of games played and the aforementioned variable, which nevertheless appears to be greater.

Almost half of the skill gamblers (44.5%) played several games, but only games involving strategy. Although gambling multi-activity constitutes a risk factor for developing pathological gambling [50], the number of games played does not seem to be the only factor to take into account. Indeed, gambling type was significantly associated with higher gambling severity when controlling for the number of games played and for psychological distress, respectively. These results suggest that mixed gambling may be a risk factor for the development of problem gambling when compared to having a purely strategic gambling activity. However, when examining size effects, our results suggest that gambling severity and gambling type are associated, but that the association may be secondary relative to the number of games played. This refers to the involvement effect, which in the literature, has been approached through the number of games played [51] and through the media used to play [52]. In this regard, Wardle and colleagues (2011) showed that gamblers using both game media (offline and online) displayed problem gambling more frequently and were more involved in the game than those using only one game medium [52]. This mixed-mode playing joins the mixed practice, as we call it in this study: gamblers have to move around to play certain games, especially for games of luck in France. Thus, the practice of mixed games, i.e., different types of games requiring the use of online and offline media, contribute more to gambling problems than the practice of playing only one type of game [32, 53]; which does not prevent the possibility of online and offline playing for the same skill game. Moreover, the practice of several games with different characteristics among the mixed gamblers (including at least one game of luck) suggests that they are probably not addicted to a particular game, but rather tend to continue their plural gambling activity more for the functions it fulfills.

The results also showed that gambling type was significantly associated with specific coping motives, suggesting that mixed gambling and coping motives are closely linked. This result is not surprising, as previous studies have highlighted that gamblers who play to escape or reduce negative affect usually move to games of luck where no reflection is required [10, 31, 36, 54]. Moreover, the association between coping motives and problematic gambling is one of the most solid results in gambling research. Indeed, the literature highlights that coping motives appear to be a strong predictor of gambling severity [3, 10, 17]. Additionally, one of the gambling disorders criteria (Diagnostic and Statistical Manual of Mental Disorders, fifth edition [DSM-5]; [1]) refers to both the gambler’s psychological distress and the coping motivation: “Often gambles when feeling distressed (e.g., helpless, guilty, anxious, depressed).” Thus, gambling appears to be a way to regulate repetitive or intrusive negative emotions among mixed gamblers. All these elements raise the hypothesis that mixed gamblers would be more likely than skill gamblers to present emotional vulnerability. Indeed, gamblers of skill games tend to play for the sensations, arousal, and excitement the game provides [10, 36, 54]. Playing these types of games can also regulate emotions, but in the sense of increasing positive feelings [36]. Problematic gambling seems to be related to emotional regulation deficit [32, 55–57]. Among skill gamblers, this seems to refer more to the presence of alexithymia [36], while among mixed gamblers, it seems to refer to difficulty in regulating negative affect efficiently and appropriately. In other words, this implies that skill gamblers have a lack of feeling and that mixed gamblers on the contrary have too many (negative) feelings. Our results as well as data from the literature indicate the importance of distinguishing these two groups of gamblers.

Strictly playing skill games, or playing both games of luck and games of skill were not associated with the inability to stop gambling. As a reminder, the perceived inability to stop gambling refers to the perceived loss of control of the gambling activity and the sense of being unable to stop it (to reduce or control it), which corresponds to one of the DSM-5 diagnostic criteria [1]. Thus, whatever the games played, with or without skills, this belief appears to be common to all gamblers with problem gambling. However, anxiety and depression seem to be related to the inability to stop gambling. The development of the belief that one is unable to stop gambling behavior can be linked to the presence of low self-esteem [56], especially in individuals who suffer from anxiety and depression.

Finally, we investigated the link between gambling type and cognitive distortions. While all gamblers are likely to develop erroneous beliefs, our study points out a difference based on the type of games played, which is consistent with previously highlighted results [58, 59]. Indeed, our results show that, when anxiety and depression are controlled for, playing only skill games significantly predicts interpretative bias. As skill games are based on strategy, experience, knowledge, and chance, it is understandable that a gambler who has done research on and has experience with the game played can attribute his successes to himself. In poker, a player who has studied the odds of a winning or losing hand can attribute a loss to bad luck after having learned that the hand in question can statistically produce a win eight times out of ten. However, because of the characteristics of these games, skill gamblers tend to overestimate their skills in the outcome and thus underestimate the part of chance. This is particularly the case in poker, where gamblers overestimate their own ability more than gamblers who play other games [58, 60]. In short, gamblers who exclusively play skill games seem to be at higher risk of misinterpreting their outcomes.

Although our findings suggest that gambling type is associated with gambling severity, coping motives, and interpretative bias, this could be explained by the fact that mixed gamblers play chance games, or the fact that they play more games, or more game types. To determine the potential impact of gambling type more precisely, our study should be replicated with gamblers who exclusively play skill games, chance games, and both types of games, while controlling for the confounding factors. However, with regards to the present results, systematically asking gamblers about the type of game they play, their gambling motives, cognitive distortions, and psychological distress can already help health professionals identify the most effective clinical interventions. One of the aims of the clinical intervention supported by our data could thus focus more on abstinence than on risk reduction, especially because the regulation of negative emotions through gambling behavior can decrease with work directly carried out on emotion regulation.

Nevertheless, this study has limitations that should be taken into account for the interpretation and generalization of the results. The main limitation is the online recruitment method (gambling forums focused on skill games) of self-selected participants, i.e., a sample that may not be fully representative of the gambling population and which contributed to the over-representation of male gamblers [61, 62]. However, the presence of only male gamblers can also be explained by the higher sex ratio of male gamblers. Indeed, the male sex represents a risk factor both in the gambling experience and the development of problematic gambling behavior [63, 64]. Moreover, female gamblers seem to present a different motivational, cognitive, and emotional profiles compared to male gamblers [14, 65, 66]. Further studies should therefore focus more specifically on female gamblers to better understand their psychological functioning with regard to gambling motives, cognitive distortions, and psychological distress. This same limitation also contributes to the absence of gamblers who exclusively play chance games and to the presence of two numerically non-homogeneous groups. Another limitation is the use of the SOGS, which is known to produce false positives, especially in the general population [67], which may explain the high prevalence of problem gamblers. Finally, certain subscales used in the present study have questionable reliability with regard to the Nunnally criteria (1978) widely used in scientific research [42]. Items within these subscales may not measure the same characteristic consistently. For example, the predictive control subscale (GRCS) assesses several types of beliefs: predictive control and probabilistic control, including the gambler’s fallacy. The same is true for interpretative bias, which is another GRCS subscale measuring attributional belief and memory bias. The low number of items can also contribute to lower internal reliability.

Despite these limitations, the results obtained support the presence of distinct psychological, motivational, and cognitive profiles according to gambling type. If the influence of the number of games played is greater, the influence of gambling type seems to exist and be at least present, requiring consideration thereof. Thus, our results suggest new research perspectives and different clinical interventions. First, it would be interesting to verify the existence of a pathological gambler typology and determine how the gambling type or the number of games played may relate to each of the three distinct subgroups of gamblers as described in the pathways model of Blaszczynski and Nower [68]. Thus, mixed gamblers with gambling problems may more frequently belong to the emotionally vulnerable group of gamblers, while skill gamblers with gambling problems may be found more frequently in the behaviorally conditioned group of gamblers. Indeed, due to the characteristics of skill games being partly governed by strategy, experience, and knowledge, gamblers continue the gambling behavior especially because they tend to underestimate the contribution of chance.

## Supporting information

S1 Dataset (XLS)
